# Rasch Analysis of the Japanese Version of Teacher Efficacy for Inclusive Practices Scale: Scale Unidimensionality

**DOI:** 10.3389/fpsyg.2020.01725

**Published:** 2020-07-29

**Authors:** Ghaleb H. Alnahdi, Akie Yada

**Affiliations:** ^1^Department of Special Education, College of Education, Prince Sattam Bin Abdulaziz University, Al-Kharj, Saudi Arabia; ^2^Department of Education, Faculty of Education and Psychology, University of Jyväskylä, Jyväskylä, Finland

**Keywords:** inclusive education, Rasch analysis, self-efficacy, Japan, TEIP

## Abstract

This study aimed to examine the construct validity of the Japanese version of the Teacher Efficacy for Inclusive Practices (TEIP) scale. The sample consisted of 250 teachers in Japan. Rasch analysis was used to examine the psychometric properties of the scale. Results did not support the 18-item Japanese version of the TEIP scale as a unidimensional scale for measuring TEIP. However, they do support the final 14-item Japanese version of the TEIP scale as a unidimensional scale for measuring TEIP. Four items were removed from the original 18-item scale (items 12, 8, 5, and 3) for violation of the local independency assumption. No item with differential item functioning (DIF) was detected. Only one item (item 18) was rescored to solve a threshold disorder. Further studies with different samples are warranted to confirm the study findings.

## Introduction

The past 30 years have seen increasingly rapid advances toward inclusive education in educational policies and systems reinforced by international policy documents (e.g., UNESCO, 1994; United Nations, 2006; United Nations General Assembly, 2015). Inclusive education can broadly be defined as including all children in mainstream classrooms regardless of their gender, their being from ethnic or linguistic minorities, or having disabilities (UNESCO, 2009). Many countries have adopted new educational strategies toward inclusive education, and Japan is no exception. A drastic change occurred in 2007 when the traditional special education (*tokushukyoiku*) system was replaced by the current special needs education (*tokubetsushienkyoiku*) system, in which children with special educational needs can officially receive appropriate support even in regular classes (Central Council for Education, 2005). Because of rapid political change toward inclusive education, Japanese teachers’ readiness to implement inclusive education has been questioned by several researchers. For instance, surveys conducted on Japanese regular classroom teachers by Ueno and Nakamura (2011) have shown that the teachers expressed anxiety and difficulty about including children with disabilities under the current support system, even though they agreed on the concept of inclusion. Similarly, Fujii (2014) studied the teachers’ awareness of keywords related to inclusive education (e.g., “reasonable accommodation” and “Convention on the Rights of Persons with Disabilities”) and suggested that the teachers’ knowledge level regarding inclusive education is relatively low and thus they need more in-service training.

Several studies on inclusive education have been conducted which report the global trend on inclusion. Investigating inclusive education from the teachers’ perspective has been one of the issues of greatest interest because they play an important role in implementing inclusive education (Avramidis and Norwich, 2002; de Boer et al., 2011). It has conclusively been shown that teachers’ self-efficacy for inclusive practices is one of the crucial factors associated with teachers’ attitudes toward inclusive education and their intention to include children with disabilities in mainstream classrooms (Savolainen et al., 2012; Sharma et al., 2018; Yada et al., 2018). The concept of self-efficacy was introduced by Bandura (1977), who defined it as a person’s belief in his/her capability to plan and execute specific performances that produce expected outcomes (Bandura, 1997). Teachers’ self-efficacy is specific to the teaching profession, and it has been found to be related not only to student achievement (Tschannen-Moran et al., 1998) but also teachers’ outcomes such as willingness to apply new teaching strategies (Allinder, 1994), and job-related stress and burnout (Betoret, 2006). Previous research has demonstrated that teachers’ self-efficacy beliefs have context-specific and goal-oriented characteristics (Wyatt, 2014), and therefore they have been studied in several teaching domains (e.g., math, language, and physical education).

Teachers’ self-efficacy has been investigated in relation to many teaching contexts; however, there was no specific instrument that assesses teachers’ efficacy beliefs in implementing inclusive education (Sharma et al., 2012). Therefore, Sharma et al. (2012) developed a scale named the Teacher Efficacy for Inclusive Practices (TEIP). The scale consists of 18 items, which can be divided into three sub-scales: (1) efficacy to use inclusive instruction, (2) efficacy in managing behavior, and (3) efficacy in collaboration (Sharma et al., 2012). The scale has been translated and used in many countries such as China, Finland, Japan, Saudi Arabia, and South Africa, and the reliability and validity of translated versions have been demonstrated (Savolainen et al., 2012; Malinen et al., 2013; Yada et al., 2018; Alnahdi, 2019b). Although there are a number of international studies focusing on teachers’ self-efficacy in inclusive practices, data about Japanese teachers’ self-efficacy are limited (Yada and Savolainen, 2017). There is the pressing need to develop a valid and reliable instrument that can measure Japanese teachers’ self-efficacy in implementing inclusive education.

Although high reliability (Cronbach’s alpha = 0.93) and good construct validity (good fit to a theoretical model of confirmatory factor analysis) were addressed for the Japanese version of the TEIP scale (Yada and Savolainen, 2017; Yada et al., 2018), these analyses were conducted according to classical test theory (CTT). In CTT, the focus is on the observed score as a whole and not on each item, such as in an item response theory like Rasch analysis (De Ayala, 2013). Rasch analysis has the benefit of providing different statistical parameters for each item. This will allow one to understand the difficulty of each item “specific perceived ability.” In the context of inclusive education, understanding the item difficulty would help to target specific teaching skills for improvement (Lai et al., 2016). The item location in Rasch analysis represents how difficult (difficult to endorse) the respective item’s “specific perceived ability” was perceived by participants, which would afford us the opportunity to pay more attention to these aspects in future training programs for teachers rather than conducting an overall assessment of teachers’ perceived self-efficacy as a whole. In addition, Rasch analysis enables us to fit the observed data to a unidimensional model, to examine the scoring structure that is being used and find the best scoring structure based on the observed data, to provide misfit persons and misfit items to be removed, and to examine measurement invariance, and it enables transformation of ordinal data into interval data (Tennant and Conaghan, 2007a; Bond and Fox, 2015). In addition, it offers the benefit of a person-item map for easy visual comparison of item difficulty and participants’ ability (Lee et al., 2010; Cappelleri et al., 2014), help to scan the scale targeting at a glance, and provide the Wright map of item-person relationship (Cheng et al., 2009). In sum, “Rasch analysis is a powerful tool for evaluating construct validity” (Baghaei, 2008b, p. 1146) that allows us to apply a unified approach to examine several measurement properties (Tennant and Conaghan, 2007a). Other reasons and advantages of using Rasch models have been documented in numerous studies (see, e.g., Andrich, 1995; Fisher, 1995, 1996; Linacre, 1996; Smith, 2000; Andrich and Marais, 2019).

Therefore, this study aims to examine the construct validity of the Japanese version of the TEIP scale using Rasch analysis. Since the total score of the TEIP scale (e.g., Ahsan et al., 2012; Miesera et al., 2019) is often used, it is important to check the scale’s unidimensionality, as “the use of the total score as a summary of a person’s value on the variable implies that persons can be compared by their total scores, or the estimates of their true scores, and this implies a unidimensional construct” (Andrich and Marais, 2019, p. 50). According to Park et al. (2016) and Lai et al. (2016) the TEIP is a unidimensional scale, and the use of the Rasch model analysis would help to examine the unidimensionality (Wright and Stone, 1999) of the Japanese version of the TEIP.

## Materials and Methods

### Sample and Instrument

The sample consisted of 250 Japanese teachers who were working in primary or secondary level (grade 1–12) schools. The data were collected using convenience sampling technique, in which schools and teachers who agreed to engage in the study were included in the sample. The sample was selected from Tokyo metropolis and eight prefectures, including Chiba, Fukui, Kagoshima, Kochi, Miyazaki, Saitama, and Yamaguchi. A sample of 250 would be needed for calibration of definitive items with 99% confidence (Linacre, 1994). The sample included 45% males and 55% females. As regards age, 36% were between 22 and 35 years old, 35% were between 35 and 50 years old, and around 29% were older than 60 years. The rate of gender and age distribution were close to those of overall Japanese teacher population (MEXT (Ministry of Education, Culture, Sports, Science and Technology), 2017).

The Japanese version of the TEIP scale was utilized to assess teachers’ self-efficacy beliefs in implementing inclusive education. The TEIP scale consists of 18 items with 6 items allocated to each sub-scale. The participants were asked to evaluate to what extent they agree/disagree with the statements on a six-point Likert scale ranging from “1 = *strongly disagree*” to “6 = *strongly agree*.” The translation of the Japanese and confirmatory factor analysis of the three factor model was confirmed in previous studies (Yada and Savolainen, 2017; Yada et al., 2018). A good level of reliability was indicated (Cronbach’s alpha = 0.93), and a good fit to the hypothesized three-factor model via confirmatory factor analysis was determined for the Japanese version of the TEIP scale (Yada and Savolainen, 2017; Yada et al., 2018).

### Rasch Analysis

In this study, the Rasch analysis steps were based on Tennant and Conaghan’s recommendations on conducting and reporting the results of a Rasch analysis study (Tennant and Conaghan, 2007a). The Rasch Unidimensional Measurement Model (RUMM2030) software (Andrich et al., 2010) was used for analysis in this study. The default model in RUMM2030, the partial credit model (Masters, 1982), is used in this study, as it is recommended with significant likelihood ratio test (Tennant et al., 2011; Vincent et al., 2015), and that means that the thresholds were estimated for each item in this type of model (Andrich and Marais, 2019). While in the rating scale model, threshold discriminations are equal across all items (Andrich, 1978).

In this analysis, we were looking for non-significant item-trait interaction chi-square as an indicator for overall fit. Another indicator would be to have normally distributed residuals with item residual mean close to zero, and a standard deviation close to 1 (Alnahdi, 2018). Threshold map and item characteristic curve (ICC) were checked for items with disordered thresholds, and any item that showed disordered thresholds was rescored to combine adjacent categories to solve this disorder (Tennant et al., 2004; Tennant and Conaghan, 2007a). We considered items with item-fit residual outside the range ±2.5 with significant *p* value to be misfit items to be removed (Tennant and Conaghan, 2007a). We considered persons outside the range of ±2.5 person-fit residual as misfit persons, to be removed from the sample (Tennant and Conaghan, 2007a). Local dependency was checked by looking for high correlation between the item residuals (Andrich and Marais, 2019). Due to the assumption that in a unidimensional scale, item residuals would not show high correlation after extracting the latent variable (self-efficacy), we considered all items with residual correlation of 0.20 above the average as an indicated violation of the local dependency assumption (Hissbach et al., 2011; Makransky and Bilenberg, 2014; Christensen et al., 2017; Lundgren-Nilsson et al., 2019). This is an important step, as it has been argued that the traditional fit statistics used in Rasch analysis could be insensitive to violations of unidimensionality under certain circumstances (Hattie, 1985; Smith, 2002; Hagquist et al., 2009).

A principal components analysis for the residuals was conducted to examine the scale unidimensionality (Smith, 2002; Tennant and Conaghan, 2007a; Andrich and Marais, 2019). Based on this analysis we got two sets of items: items that loaded positively on the first component and items that loaded negatively on the first component. Then, two ability estimates were calibrated based on these two sets of items. Next, *t*-tests were conducted to examine whether the two ability estimates were statistically significant. A 5% or less significant test would be considered acceptable or the lower limit of 95% for the binomial proportion confidence intervals at 5% level or less (Smith, 2002; Tennant and Conaghan, 2007a; Alnahdi, 2018).

We examined Differential item functioning (DIF) for items to ensure that items function similarly for both gender and regardless of participants’ age (Tennant and Pallant, 2007b). We examined internal consistency looking for the value of the person separation index (PSI) >0.7 as a good indicator (Tennant and Conaghan, 2007a). Finally, we transformed the raw scores to interval scores using the formula: “Y = M + (S × logit score). S = range of interval-level scale [(60; for a 0–60 scale)] divided by the actual range of logit scores, and M = (minimum score of interval-level scale) – (minimum logit score × S)” (Alnahdi, 2018, p. 355). This step made the interpretation of the scores much easier because any change in one unit would have the same weight across scale (Alnahdi, 2018).

## Results

In the first analysis, we examined the 18-items scale fit to the Rasch model. Table 1 shows that chi-square for item-trait interaction was non-significant [χ^2^ 78.05 (72) = *p* > 0.05], which was a good indicator. However, the results did not support the scale unidimensionality as 14% of *t*-tests were significant in comparison with the recommended limit of 5% (see Table 1).

**TABLE 1:** Rasch statistics at each run.

M			Item residual fit		Person residual fit		Item-trait interaction			Unidimensionality *t*-tests	
		*N*	Mean	*SD*	Mean	*SD*	χ ^2^ (*df*)	*p*	PSI	% significant tests	Lower limit of 95% CI^*a*^
A	Initial analysis	250	0.25	1.43	−0.32	1.44	78.05 (72)	0.292	0.933	14%	9.94%
	After removing 18 misfit persons	232	0.39	1.75	−0.22	1.27	97.63 (72)	0.023	0.937	13.8%	9.63%
	After rescoring one item (18)	232	0.39	1.75	−0.22	1.27	97.68 (72)	0.023	0.936	13.8%	9.63%
	4 super items *	232	0.45	1.39	−0.19	1.20	88.08 (56)	0.003	0.928	12.9%	8.9%
	After removing item 12	232	0.37	1.82	−0.22	1.25	93.07 (68)	0.023	0.933	13.8%	9.63%
	After removing item 8	232	0.37	1.55	−0.21	1.21	92.15 (64)	0.012	0.927	13.8%	9.63%
	After removing item 5	232	0.30	1.31	−0.22	1.19	96.34 (60)	0.002	0.921	9.91%	6.38%
	After removing item 3	232	0.29	1.29	−0.22	1.18	67.51 (56)	0.139	0.919	7.76%	**4.46%**
B	EMB subscale	232	−0.12	1.88	−0.40	1.12	47.78 (24)	0.002	0.870	11.6%	7.8%
	EII subscale	232	0.09	0.62	−0.35	1.06	17.84 (24)	0.810	0.855	9.5%	6%
	EC subscale	232	0.24	1.21	−0.27	0.98	22.78 (24)	0.532	0.830	8.2%	**5%**

Bold = indicator of unidimensionality; a = the lower limit of 95% for the binomial proportion confidence intervals, M = models, A = unidimensional, B = multi-dimensional, EMB = efficacy in managing behavior, EII = efficacy in inclusive instruction, EC = efficacy in collaboration, four items that showed local independency indictors composite together, * = after removing two items, the remaining two items (item 3 with item 9 and item 5 with item 6) that showed indicators of local independence are combined.

**FIGURE 1:**
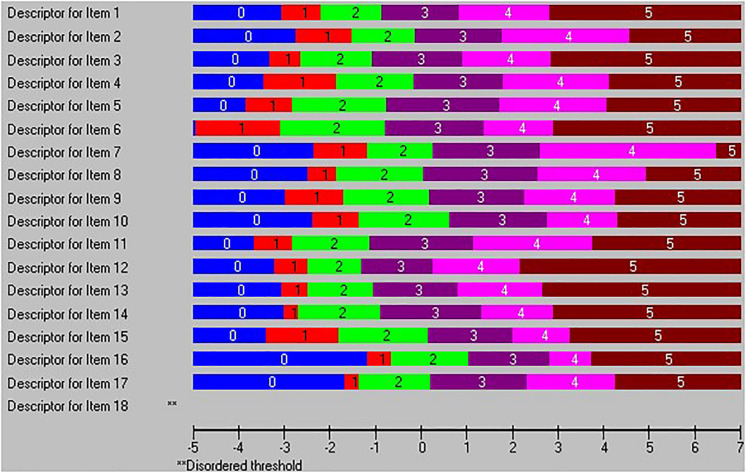
Threshold map indicating that item 18 has threshold disorder.

Next, in the second run, 18 misfit persons with residuals outside the ±2.5 range were removed. As shown in Table 1, the unidimensionality issue was still not solved by this modification, and the percentage of significant *t*-tests decreased only to 13.8%, which was still far above the recommended 5%. Next, we examined the threshold map looking for items with disordered threshold. Only item 18 was found to have threshold disorder (see Figure 1).

**FIGURE 2:**
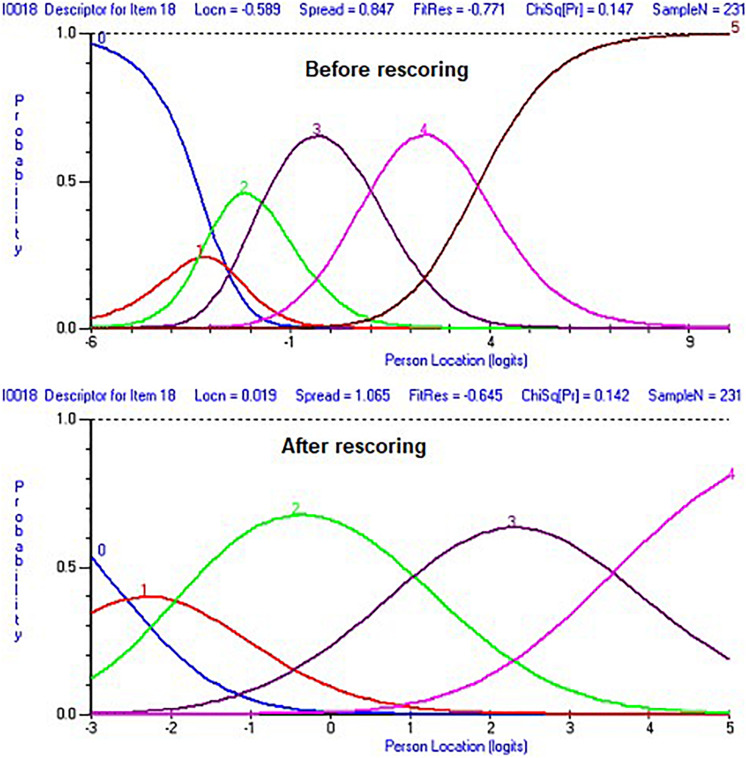
Category probability curves for item 18 before and after rescoring the item.

In the next run, item 18 was rescored to improve the threshold order, and instead of the previous score of 012345, it became 001234. This new score improved the threshold order (see Figure 2). However, there was no improvement with regards to the unidimensionality test. Next, we examined local dependency of items by reviewing residuals correlation of items, looking for a value of 0.20 or higher. Four items were found to have that level of correlation with other items.

Next, to overcome local dependency, the four items whose residuals showed high correlations with other item residuals were combined into four super items (Marais and Andrich, 2008) (item 8 with item 7, item 12 with item 13, item 5 with item 6, item 3 with item 9) to check whether this improved scale unidimensionality. This is recommended as a solution, for “when a set of items are locally dependent they can be bundled into polytomous super-items” (Baghaei, 2008a, p. 1105), and it has been applied in several studies (such as Marais and Andrich, 2008; Brogårdh et al., 2013; Peter et al., 2014; Medvedev et al., 2016; Milinis et al., 2017; Finger et al., 2019). However, this step did somewhat improve the percentage of significant *t*-tests in the unidimensionality test, 12.9%, though this is still far from the recommended 5%.

In the next four runs, we removed these items one by one and examined improvement in the unidimensionality test. In the last run, after removing the four items, the unidimensionality was supported by finding that the lower limit of the 95% CI for the binominal test was less than 5% (4.46%). The removed items were from all three subscales: item 8 was from efficacy in managing behavior; item 5 was from efficacy in inclusive instruction subscale; and items 3 and 12 were from efficacy in collaboration subscale. Since the scale showed unidimensionality and had no items with high residual correlations, the assumption of the local independence of items was fulfilled (Tennant and Conaghan, 2007a) in the final 14-item Japanese TEIP scale.

In addition, as an alternative to removing the four items, three extra runs were conducted separately for each subscale to see whether the scale would show better fit as a multidimensional scale with three unidimensional subscales. The results showed that only the efficacy in collaboration subscale fit the Rasch model and the unidimensionality test supported a unidimensional subscale. However, the results did not support the unidimensionality of the other two subscales. Therefore, we continued with the 14 items as a unidimensional scale in further analysis.

**FIGURE 3:**
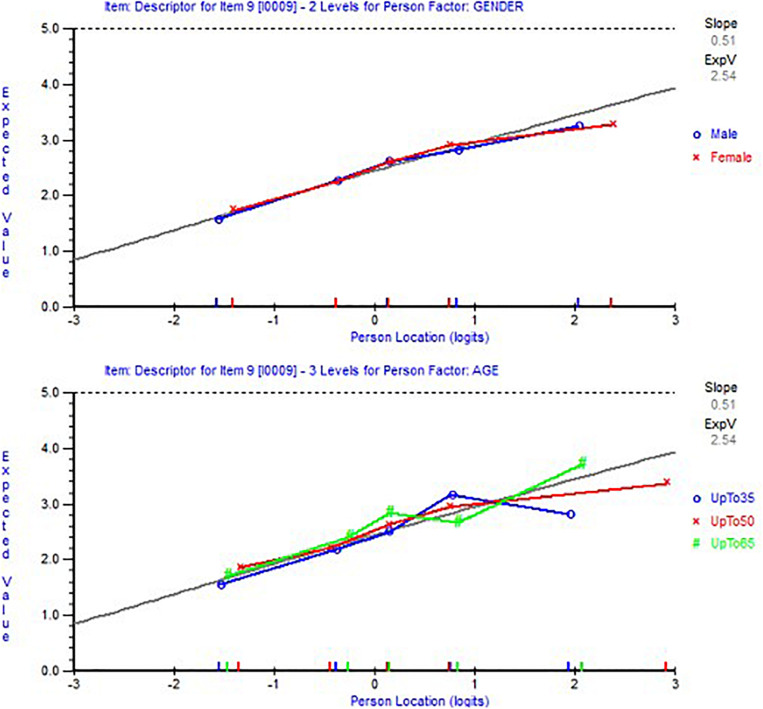
Item characteristic curves showing an example of item 9 with no differential item functioning (DIF) by age (bottom figure) nor by gender (top figure).

After we ensured the unidimensionality of the 14-item scale, a DIF analysis was conducted to ensure the 14 items function similarly regardless of the sample age or gender. The results indicated that no item showed indicators of DIF. Figure 3 shows an example of item 9 that functioned similarly regardless of participants’ age or gender. Table 2 shows item parameters, and items were sorted based on location, from most to least difficult to comply with the lowest location value.

**TABLE 2:** Item fit statistics sorted based on location, from the most to the least difficult item to endorse.

Item	Location	*SE*	Fit residual	χ ^2^	*p*^*a*^
7	1.673	0.089	0.653	1.106	0.893
16	1.097	0.082	3.212	2.216	0.696
17	0.845	0.087	−0.769	3.616	0.460
10	0.659	0.093	−1.547	12.1	0.016
2	0.200	0.092	−0.501	2.27	0.686
9	0.191	0.092	0.837	7.135	0.128
4	0.033	0.093	−0.775	1.752	0.781
15	−0.036	0.088	0.061	4.15	0.386
18	−0.036	0.104	−0.829	5.519	0.238
1	−0.662	0.086	2.569	9.061	0.059
11	−0.757	0.104	0.007	4.385	0.356
13	−0.827	0.089	0.396	8.146	0.086
6	−1.109	0.097	0.445	1.577	0.812
14	−1.271	0.095	0.352	4.484	0.344

*SE, standard error. ^*a*^ Bonferroni adjusted *p* = 0.00357 (0.05/14).*

Table 3 shows the scale with the new scoring applied. One item (18) was rescored as 001234, and the remaining 13 items were not changed and they were 012345. Table 4 shows the transformation of raw scores to interval scores, which will make it easier to interpret any differences in interval scores, because any change in one unit has the same weight across the scale (Alnahdi, 2018).

**TABLE 3:** The final Rasch-validated scale with 14 items.

*Item*		Strongly disagree	Disagree	Disagree somewhat	Agree somewhat	Agree	Strongly agree
1	I can make my expectations clear about student behavior	0	1	2	3	4	5
2	I am able to calm a student who is disruptive or noisy	0	1	2	3	4	5
4	I can assist families in helping their children do well in school	0	1	2	3	4	5
6	I can provide appropriate challenges for very capable students	0	1	2	3	4	5
7	I am confident in my ability to prevent disruptive behavior in the classroom before it occurs	0	1	2	3	4	5
9	I am confident in my ability to get parents involved in the school activities of their children with disabilities	0	1	2	3	4	5
10	I am confident in designing learning tasks so that the individual needs of students with disabilities are accommodated	0	1	2	3	4	5
11	I am able to get children to follow classroom rules	0	1	2	3	4	5
13	I am able to work jointly with other professionals and staff (e.g., aides, other teachers) to teach students with disabilities in the classroom	0	1	2	3	4	5
14	I am confident in my ability to get students to work together in pairs or in small groups	0	1	2	3	4	5
15	I can use a variety of assessment strategies (e.g., portfolio assessment, modified tests, performance-based assessment)	0	1	2	3	4	5
16	I am confident in informing others who know little about laws and policies related to the inclusion of students with disabilities	0	1	2	3	4	5
17	I am confident when dealing with students who are physically aggressive	0	1	2	3	4	5
18	**I am able to provide an alternative explanation or example when students are confused**	**0**	**0**	**1**	**2**	**3**	**4**
**Removed Items (below)**							
3	I can make parents feel comfortable coming to school						
5	I can accurately gauge student comprehension of what I have taught						
8	I can control disruptive behavior in the classroom						
12	I can collaborate with other professionals (e.g., itinerant teachers or speech pathologists) in designing educational plans for students with disabilities						

*Boldface = rescored items.*

**TABLE 4:** Transformation table for the conversion of the 14-item Japanese TEIP Scale total raw ordinal-level score to interval-level score.

Raw score	Interval-level score	Raw score	Interval-level score	Raw score	Interval-level score	Raw score	Interval-level score
0	0.0	18	17.1	36	24.6	54	33.7
1	3.4	19	17.5	37	25.0	55	34.3
2	5.7	20	17.9	38	25.5	56	34.8
3	7.4	21	18.3	39	26.0	57	35.5
4	8.6	22	18.7	40	26.4	58	36.1
5	9.7	23	19.1	41	26.9	59	36.8
6	10.5	24	19.5	42	27.4	60	37.5
7	11.3	25	19.9	43	27.9	61	38.2
8	12.0	26	20.3	44	28.4	62	39.1
9	12.6	27	20.7	45	28.9	63	40.0
10	13.2	28	21.1	46	29.4	64	41.1
11	13.8	29	21.5	47	29.9	65	42.3
12	14.3	30	22.0	48	30.5	66	43.9
13	14.8	31	22.4	49	31.0	67	46.0
14	15.3	32	22.8	50	31.5	68	50.5
15	15.7	33	23.2	51	32.0	69	60.0
16	16.2	34	23.7	52	32.6		
17	16.6	35	24.1	53	33.1		

**FIGURE 4:**
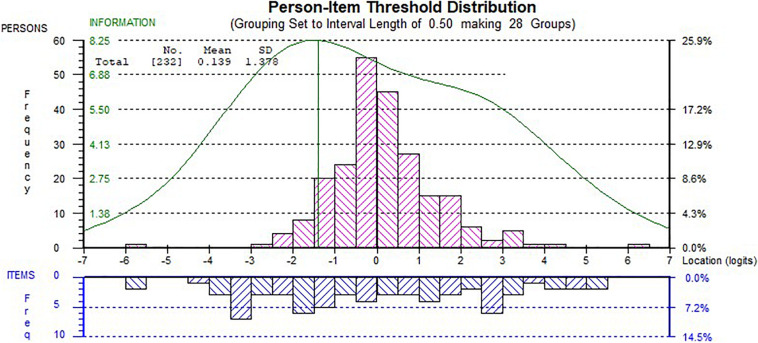
Person-item threshold plot of the 14-item scale. Distribution of teachers’ ability estimates (top) and item thresholds (bottom). The curve represents the information function of the scale.

In addition, we examined the internal consistency of the 14-item scale, and the person separation index (PSI) was 0.91 which indicated a high level of internal consistency as the adequate value is 0.70 (Tennant and Conaghan, 2007a). Figure 4 shows the person-item threshold plot with good spread of item thresholds covering the partisan threshold as a good indicator for targeting.

## Discussion

This study aimed to examine the construct validity of the Japanese version of TEIP using Rasch analysis. The Rasch analysis of the 18-items scale did not support a unidimensional scale. This result is not consistent with the result of Park et al. (2016) via confirmatory factor analysis that the 18-item TEIP is a unidimensional scale. However, Lincacre argued that “there is no way of knowing from factor analysis alone whether each factor is a dimension or a slice of a shared dimension” (Linacre, 1998, p. 603). This result is consistent with the findings of another Rasch study of the Arabic version of the scale (Alnahdi, 2019a) that did not support the unidimensionality of the 18-item TEIP. This shows the importance of more combined studies with data from different countries to examine the cross-cultural psychometric properties of the scale.

Modifications were made to improve the fit for the Rasch model; removing misfit persons, rescoring one item (18) for threshold disorder, and removing four items for violating the local independency assumption. After these changes took place, the 14-item scale did fit the Rasch model and support a unidimensional scale.

The four items that were removed were items 3, 5, 8, and 12. If we look at these items, which have high correlated residuals, to understand the source of the local dependency issue, we see that the residual of item 12 “*I can collaborate with other professionals (e.g., itinerant teachers or speech pathologists) in designing educational plans for students with disabilities*” was correlated with residual from item 13 “*I am able to work jointly with other professionals and staff (e.g., aides, other teachers) to teach students with disabilities in the classroom.*” In these two items, 12 and 13, it appears that there is a level of repetition of the collaborative idea that might cause a local dependency issue of items, as more redundant items could increase the dependence in the data (Marais and Andrich, 2008). A similar observation was made with item 8 “*I can control disruptive behavior in the classroom*” as its residual was correlated highly with the residual from item 7 “*I am confident in my ability to prevent disruptive behavior in the classroom before it occurs*,” and for residuals for items 5 and 6, and items 3 and 9. Moreover, it is important that we deal with items with local dependency as “dependency among items can inflate reliability and give a false impression of the precision and quality of the test” (Baghaei, 2008a, p. 1105). Hattie (1985) believes that “the most critical and fundamental assumption of the latent trait models is that of local independence” (p. 151), and Wang et al. (2005) also discussed the misspecification that can be a result of violating the local item independence assumption.

In addition, before we removed the four items, we tested the super-item solution, which did not solve the problem of local dependency in the 18-item TEIP scale unidimensionality. Therefore, we continued by removing four items and proposed the 14-item scale with as a unidimensional measure, as the “violations of the unidimensionality requirement influence person measurement” (Smith, 2002, p. 205). In line with this, Clark and Watson discussed the issues related to scales construct validity, and they stated that “in selecting scale items, the goal is unidimensionality rather than internal consistency” (p. 306, Clark and Watson, 1995). Two of the four items removed in this study, items 8 and 12, were also removed from the Arabic version of the scale to reach a unidimensional scale (Alnahdi, 2019a) for the same reason, the violation of the local item independence assumption.

We found that item 7 “*I am confident in my ability to prevent disruptive behavior in the classroom before it occurs*” was the most difficult item to be endorsed by participants, followed by items 16, 17, and 10. These results are consistent with those of Yoshitoshi (2014) who administered the TEIP scale to 59 high school teachers in Japan. He indicated that Japanese teachers reported more negative responses to items 7 and 17 and concluded that teachers did not know of concrete strategies to work with children who show difficult behavior. Further, item 16 is related to knowledge about laws and policies of inclusive education and this was the second difficult question for the Japanese teachers to endorse. This finding is somewhat in line with that of a previous study indicating that Japanese teachers showed less awareness of the keywords related to inclusive education than those related to special needs education (Fujii, 2014). These findings have important implications for developing in- and pre-service teacher training in Japan, where teachers can acquire necessary skills to deal with students’ problematic behavior and knowledge regarding inclusive educational laws and policies. The easiest item to be endorsed was item 14 followed by items 6, 13, and 11. Items 11 and 14 were among the consistently weakest indicators for self-efficacy for this sample and for the Saudi sample studied earlier (Alnahdi, 2019a). Similarly, Chao et al. (2018) found item 11 to be convenient for endorsement in Hong Kong (“*I am able to get children to follow classroom rules*”).

The final 14-item scale that fit the Rasch model was different from the 13-item scale that fit the Rasch model with a sample from Saudi Arabia (Alnahdi, 2019a). This shows that items are perceived somewhat differently in different populations for different reasons. Therefore, it would be recommended for future studies to have combined data from different countries to examine the unidimensionality of the TEIP scale for different populations. Also, this will allow one to examine if all items function similarly in different countries. It will also allow examining the best scoring structure of the scale, as this scale has been used with different scoring structures in different studies with 5, 6, and 9-point Likert scales (Lai et al., 2016; Park et al., 2016; Yada et al., 2018; Alnahdi, 2019a).

## Implications

As mentioned above, developing inclusive education is on the global educational agenda to realize an inclusive society (United Nations General Assembly, 2015). Thus, there are increasing needs to examine continuously whether the new inclusive educational policies and systems are operated well in practice. One way for that is to measure teachers’ self-efficacy for inclusive practices, which reveals the teachers’ perspectives on inclusive education. Our findings confirmed that the 14-item Japanese version of the TEIP scale is valid to assess Japanese teachers’ self-efficacy for inclusive practices and can be used not only by researchers but also by government and municipal administrators. In addition, this finding will help by providing researchers with a clear order of the items in the scale, for items with strength indicator for teachers’ efficacy, and this would be helpful in cases where researchers would need to use only a few items as part of their studies. So, items with highest value on location can be chosen. Understanding tasks (items) order would help to arrange tasks that included in TEIP according to the difficulty level from the teachers’ point of view, which is important in designing curricula and training programs for teachers to focus more on the difficult tasks. In addition, it will be helpful to measure improvements as a result of intervention studies. Transformation tables of scores help researchers to easily interpret differences in scores and determine how significant a change is in some score after receiving an intervention, for example. The findings of this study would support researches to calculate a total score on the 14-item Japanese TEIP to represent a teacher’s efficacy to work in inclusive education. Finally, further research is required to confirm the findings of this study.

## Data Availability Statement

The datasets generated for this study are available on request to AY, ado.eika@gmail.com.

## Ethics Statement

Ethical review and approval was not required for the study on human participants in accordance with the local legislation and institutional requirements. The patients/participants provided their written informed consent to participate in this study.

## Author Contributions

All authors listed have made a substantial, direct and intellectual contribution to the work, and approved it for publication.

## Conflict of Interest

The authors declare that the research was conducted in the absence of any commercial or financial relationships that could be construed as a potential conflict of interest.
